# Surgical resection of retrohepatic inferior vena cava leiomyosarcoma without vascular reconstruction: case report

**DOI:** 10.1590/1677-5449.202201081

**Published:** 2023-07-10

**Authors:** Ian Freire Castro, Paulo Henrique Silva Nunes, Ana Camila Xavier Lopes, Mariana Coelho Lima, Régis Ponte Conrado, Renato Mazon Lima Verde Leal, Annya Costa Araújo de Macedo Goes, Marcelo Leite Vieira Costa

**Affiliations:** 1 Hospital Geral de Fortaleza - HGF, Fortaleza, CE, Brasil.; 2 Universidade Federal do Ceará - UFC, Fortaleza, CE, Brasil.; 3 Hospital Universitário Walter Cantídio - HUWC, Fortaleza, CE, Brasil.

**Keywords:** leiomyosarcoma, vena cava inferior, retroperitoneal neoplasms, sarcoma, vascular surgical procedures

## Abstract

Inferior vena cava leiomyosarcoma (IVCL) is a rare malignant mesenchymal tumor. Surgical treatment is a challenge because it must combine free surgical margins with vascular reconstruction, using prosthetic or autologous grafts, primary suture, or simple ligation without vein reconstruction. The ligation option is possible thanks to the slow growth of the tumor, allowing collateral venous circulation to develop. We present a case of an IVCL treated with radical resection without vascular reconstruction. The patient was a 48-year-old female with abdominal pain in the right upper quadrant, asthenia, and postprandial dyspeptic symptoms. Abdominal tomography revealed a mass with an expansive formation located in the infrahepatic segment of the inferior vena cava and reduced vessel lumen. During surgery, vein clamping did not provoke hemodynamic repercussions, suggesting sufficient collateral circulation formation. It was decided to perform a radical resection of the entire portion of the retrohepatic vena cava and ligate the vena cava without vascular reconstruction. The patient recovered without complications.

## INTRODUCTION

Inferior vena cava leiomyosarcoma (IVCL) is a rare malignant mesenchymal tumor originating in venous smooth muscle. Since it was described for the first time by Perl in 1871, only just over 400 cases have been documented worldwide.^1^ Around 2% of all leiomyosarcomas occur in major vessels, 60% of which are found in the inferior vena cava (IVC).^2^ Since they grow slowly, they remain asymptomatic for a long time and are diagnosed accidentally in 10.5% of cases.^3^

Habitually, IVCL produces late and nonspecific clinical manifestations. Symptoms depend on which segment of the vein is affected. Mild and nonspecific abdominal pains are the most prevalent symptom and rarely a palpable abdominal mass may be found during physical examination. Women are affected more frequently and incidence peaks in the sixth decade of life. Presentation at diagnosis is as a large tumoral mass, with a mean size of 10.8 cm.^4^ Surgical resection of an IVCL is challenging because it is necessary to achieve free surgical margins in combination with vascular reconstruction, using a prosthetic or autologous graft, primary suture, or simple ligature without reconstruction of the vena cava.^2,5^

This article was approved by the Research Ethics Committee, consolidated opinion number 5.560.148 (CAAE: 59402022.6.0000.5045).

## CASE REPORT

**Figure 1:**
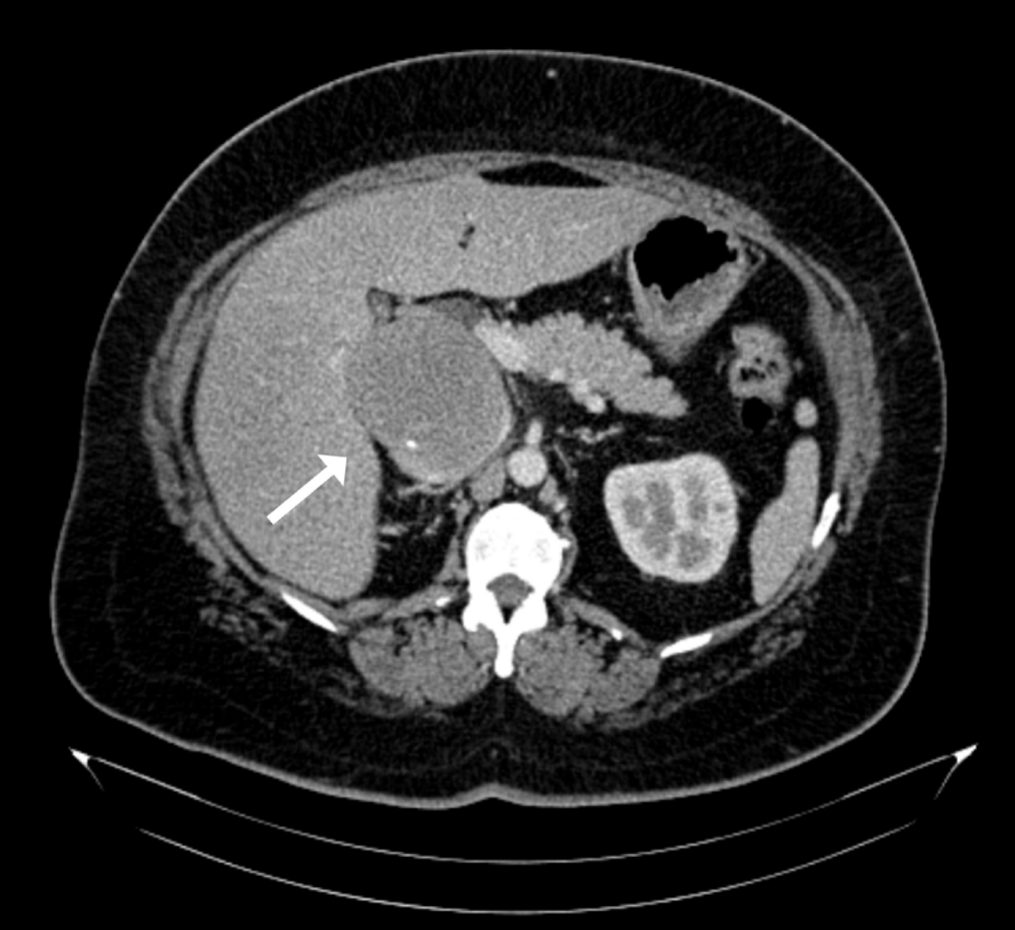
Computed tomography image of the abdomen from the arterial phase, showing the tumor exerting mass effects on the liver and considerable reduction of the lumen of the retro-hepatic vena cava.

**Figure 2:**
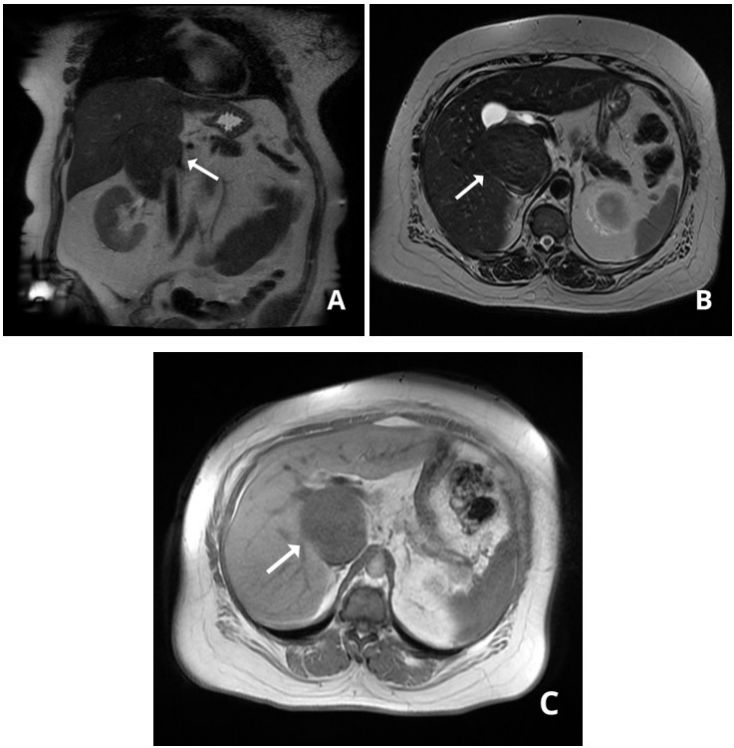
(a) Coronal magnetic resonance image in T2 showing the leiomyosarcoma exerting a mass effect on the area of the hepatic hilum and obstructing the inferior vena cava; (b) T2 axial image showing the lesion with regular outlines and well-defined limits, with low homogenous signal, and compressing the vena cava; (c) dual echo axial image showing the leiomyosarcoma in contact with segments I, V, and VI of the liver.

A forty-eight-year old previously healthy housewife sought medical attention in 2016 for dyspeptic symptoms that worsened postprandially, abdominal pains in the right hypochondrium, and asthenia. She had no family history of cancer. Physical examination found her in good general health, with abdominal pain in response to palpation and edema of the lower limbs. Abdominal ultrasonography (USG) showed a retro-hepatic mass with well-defined margins. Having abandoned outpatients follow-up, she was referred to our service in 2019. Her painful and dyspeptic symptoms had worsened, but she no longer had edema of the lower limbs. Abdominal USG showed a lesion with well-delineated anechoic content in the hepatorenal space, measuring 10.5 x 7.3 cm. Abdominal computed tomography (CT) showed an expansive mass located in the retroperitoneal space, in the infra-hepatic segment of the IVC, with considerable reduction of the lumen (Figure 1). A CT-guided liver biopsy indicated a low histological grade spindle cell tumor. Immunohistochemistry identified it as a smooth muscle tumor of uncertain malignant potential (STUMP). Magnetic resonance showed that its dimensions were stable in relation to previous examinations (Figure 2).

**Figure 3:**
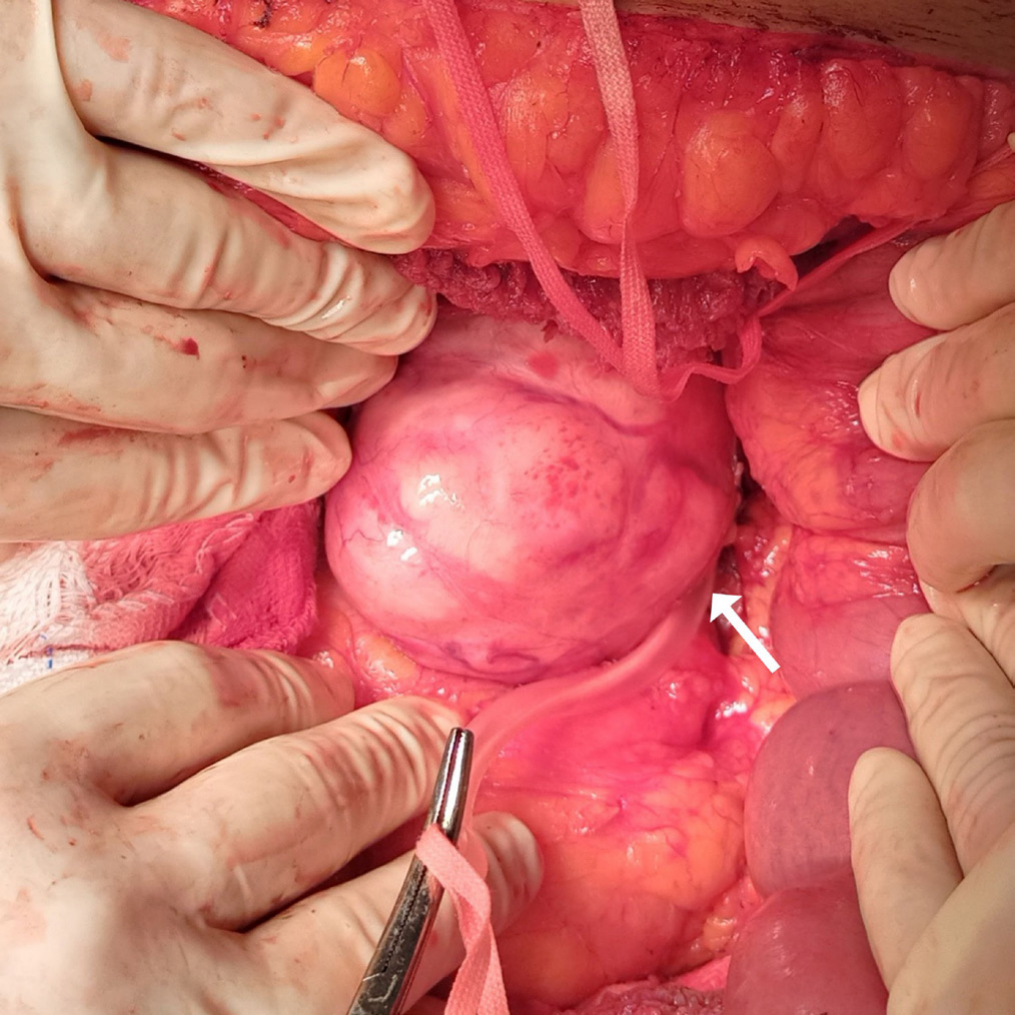
View of the leiomyosarcoma after full mobilization of the liver and before clamping the vena cava above and below the tumor.

**Figure 4:**
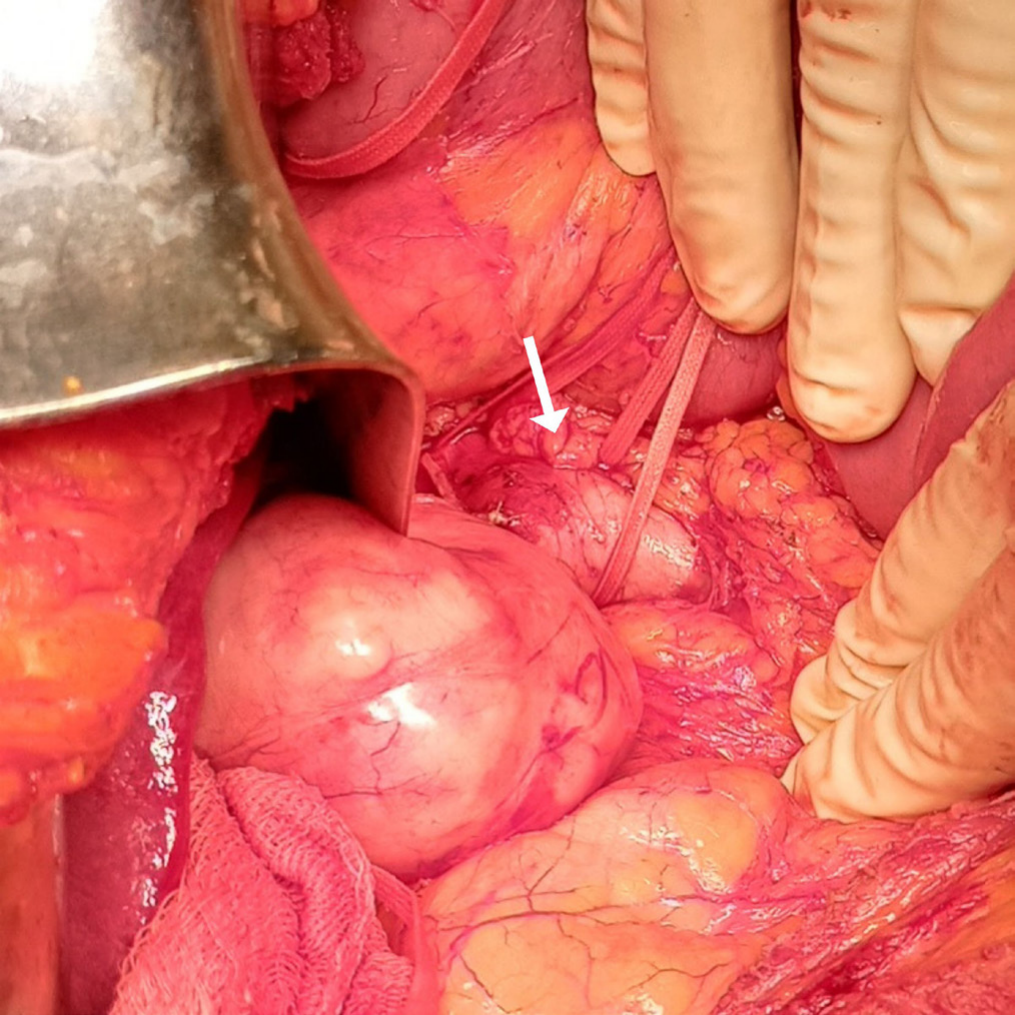
Intraoperative image showing the inferior vena cava (arrow) and the leiomyosarcoma (on the left) before surgical resection.

**Figure 5:**
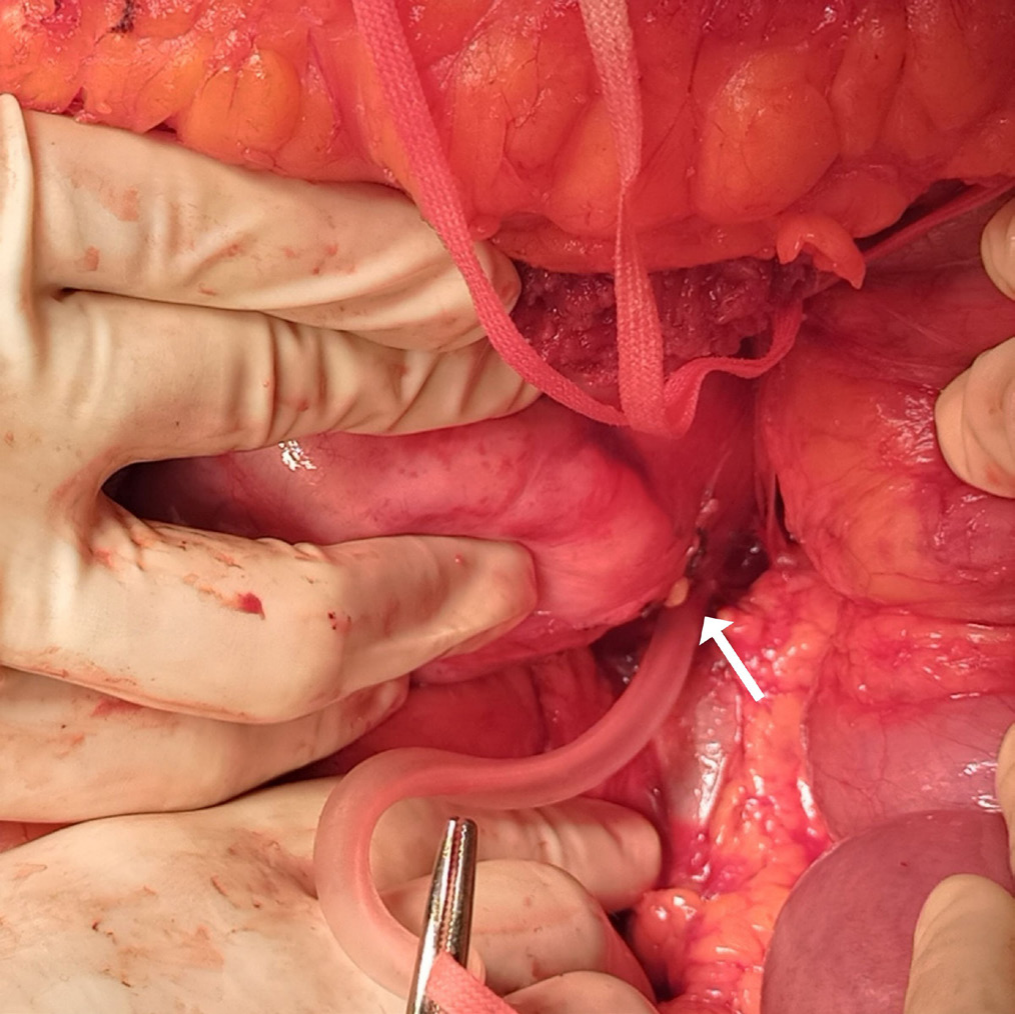
Exeresis of the inferior portion of the tumor and hemostasis with vascular staples (arrow).

**Figure 6:**
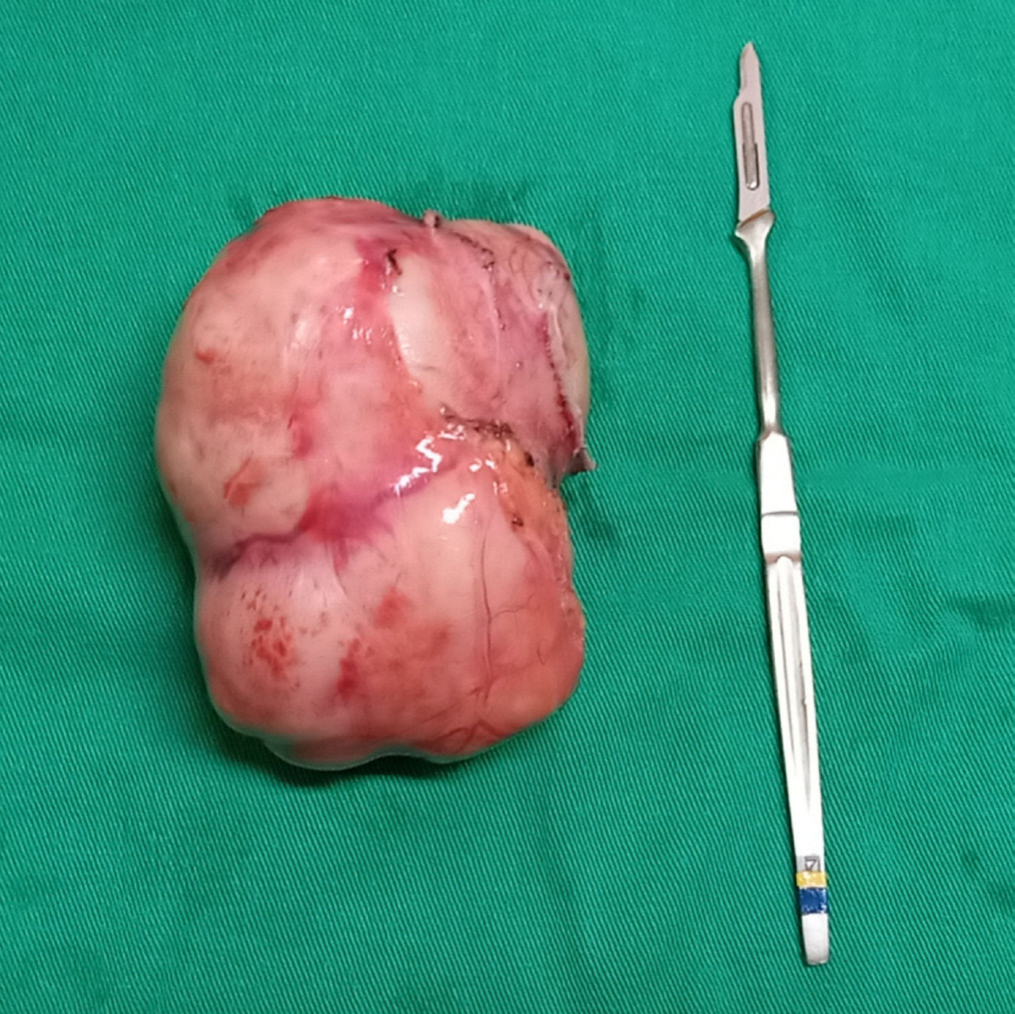
The leiomyosarcoma after surgical resection.

Surgery was performed in May of 2021. Access was obtained via a transverse bilateral incision combined with a complete Kocher maneuver and extensive mobilization of the right lobe of the liver, exposing the entire extent of the abdominal cava, from the diaphragm to the renal veins (Figure 3). The tumor was visible in the retro-hepatic portion, obstructing the lumen of the vessel, but without invasion of the hepatic parenchyma or adjacent organs (Figure 4). After clamping the vein, no hemodynamic repercussions were observed, indicating that sufficient collateral circulation had formed in response to the chronic obstruction. It was decided to resect the tumor in monobloc with the entire retro-hepatic portion of the vena cava (Figure 5). The stumps of the IVC were sutured with vascular staples and no vascular reconstruction of the sectioned segment was attempted. The postoperative anatomopathological examination revealed well-differentiated leiomyosarcoma, measuring 9.5 x 7.0 x 6.5 cm and weighing 281 g (Figure 6), with type 1 histological grade. No genetic analysis of the surgical specimen was conducted because this type of test is not available at our institution.

**Figure 7:**
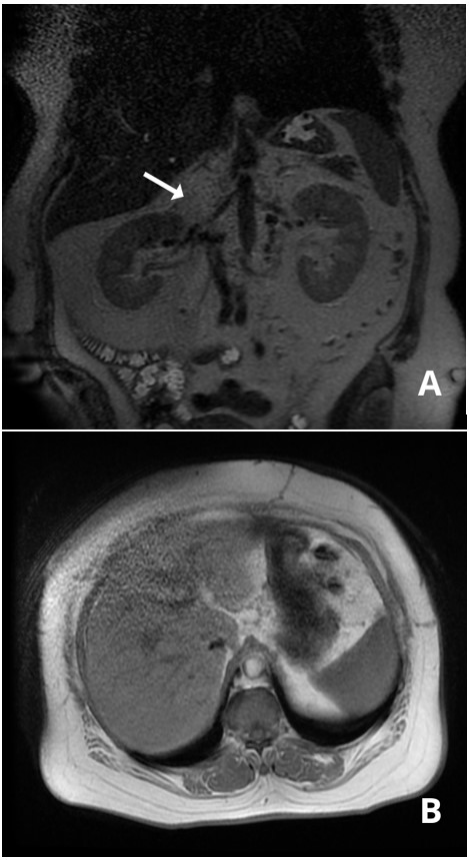
(a) Coronal magnetic resonance image in T2, 17 months after surgery, showing the sectioned segment of the inferior vena cava and absence of local relapse; (b) dual echo axial image showing the retro-hepatic region tumor free after surgery.

The patient recovered with no intercurrent conditions and was discharged 5 days later. Seventeen months after surgery, she was stable and free from local or remote relapses (Figure 7). She is attending six-monthly follow-ups at our service.

## DISCUSSION

Anatomically, IVCL can be classified into three groups: segment I (infrarenal), segment II (between the renal veins and the hepatic veins), and segment III (supra-hepatic).^2,6^ Tumors in segment II, as described in the present report, are the most common type and have the best prognosis.^6^ They generally only manifest with pain in the right hypochondrium, but can mimic diseases of the biliary tract and if the renal vessels are involved they can cause kidney failure.^2^

A precise diagnosis is made with histopathological examination of a biopsy taken under USG or CT guidance.^1,7^ It is important to assess differential diagnoses of IVCL, which include tumors that invade the IVC, such as renal cell carcinoma, and primary retroperitoneal neoplasms, such as leiomyomas, adipocyte tumors, fibroblastic tumors, rabdomiosarcomas, and cystic tumors.^7^

It is essential to perform a complete circumferential resection of the IVC with negative margins to minimize the risk of local recurrence.^2,4^ The tumor generally adheres loosely to the adjacent structures and is limited to its site of origin, enabling it to be dissected with relative ease. In these cases, radical resection does not need more than 1 cm of free vena cava margin.^8^ If adjacent organs are involved, it is necessary to resect the involved segments.^2^

Options for reconstruction of the IVC include primary repair, autologous graft, prosthetic graft, or simple ligature without reconstruction.^9^ Ligature of the vein is possible thanks to the slow growth of the tumor over the course of months or years, enabling development of collateral venous circulation.^10^ Large en bloc resections may disrupt the collateral venous network, making ligature of the vein impossible.^11^ Ligature reduces the duration of surgery and avoids the complications associated with repair by prosthetic grafting, such as hemorrhages, infections, and the need for anticoagulants. The principal risks of ligature of the IVC include chronic venous insufficiency of the lower limbs, renal failure, and edema of the lower limbs.^2^

It is essential to define which patients will benefit or not from ligature of the IVC. To this end, Jiang et al.^12^ proposed criteria to for indicating ligature of the cava based on the experience at their center: (1) if disease onset is more than 1 year previously and so sufficient collateral circulation may have developed; (2) if at least 75% of the IVC is obstructed; and (3) if an intraoperative intravenous injection of 20 mg of furosemide provokes more than 100 mL of urine within 30 minutes after temporary blockage of the IVC. Another method for assessing the need to reconstruct the IVC is to measure venous pressure in the IVC after clamping. Reconstruction may be necessary if pressure exceeds 30 mmHg.^11^ Presence of edema of the lower limbs at physical examination is an indication of insufficient collateral venous formation and makes IVC reconstruction obligatory.^2^

According to Daylami et al.,^13^ good clinical results can be achieved without IVC reconstruction in tumors that develop below the hepatic veins. In their study with six patients, four had their IVC occluded by the tumor. None of the patients had IVC reconstruction. After surgery, three patients developed edema of the extremities, which was well-tolerated and caused no permanent sequelae. None of the patients had local relapse during the 30-month follow-up.^13^ Hollenbeck et al.^14^ published a retrospective study with 25 patients, 52% of whom were treated with ligature of the IVC after resection of the tumor. The most common postoperative morbidity was edema of the lower limbs, but none of the patients had persistent severe edema. Ligature of the IVC above the renal veins was well-tolerated in patients with good preoperative renal function and with suprarenal thrombosis or venous obstruction and formation of collateral circulation.^14^

Studies show that perioperative radiotherapy is related to increased global survival when compared with surgery alone in cases of retroperitoneal sarcoma. Adjuvant radiotherapy was also associated with increased survival in specific studies of IVCL and can be used to reduce the likelihood of local relapse in patients with positive surgical margins. Neoadjuvant chemotherapy and radiotherapy can be useful to increase the chance of surgical resection for tumors that are unresectable. In these situations, the chemotherapy drugs most often used are dacarbazine, doxorubicin, cyclophosphamide and iphosfamide/cisplatin. However there is no standard chemotherapy regimen for IVCL and the evidence for its use is contradictory. Currently, investigations are ongoing into use of insulin-like growth factor 1 blockers, mammalian target of rapamycin inhibitors, and poly ADP-ribose polymerase inhibitors.^3,7,9^

After resection of the tumor, survival rates at 5 and 10 years are 49.4% and 29.5%, respectively, and 5-year disease-free survival is 6%.^9^ Local recurrence is observed in up to 53% of cases and the most frequent sites of metastases are the liver, lungs, and bones.^4^ Prognosis is strongly linked to the extent of radical resection, presence of negative surgical margins, the size of the tumor, and absence of metastases at the time of surgery.^7,9^ In cases with relapse, surgery continues to be an important treatment option, and can improve survival. In cases in which complete resection is impossible, palliative treatment can be based on cytoreduction combined with radiotherapy.^1^

## CONCLUSIONS

Inferior vena cava leiomyosarcomas are rare malignant neoplasms with nonspecific clinical presentation. Diagnosis is made with imaging exams and confirmed by biopsy. Radical surgical resection is the only treatment that offers a possibility of cure. In selected cases it is unnecessary to reconstruct the vena cava with prostheses or grafts, because of formation of collateral venous circulation that enables simple ligature of the vein to be performed safely. The role of neoadjuvant and adjuvant treatments is not yet well-established and further studies are needed to determine the most appropriate treatment.

## References

[1] Rusu CB, Gorbatâi L, Szatmari L (2020). Leiomyosarcoma of the inferior vena cava. Our experience and a review of the literature. Rom J Morphol Embryol.

[2] Gaignard E, Bergeat D, Robin F, Corbière L, Rayar M, Meunier B (2020). Inferior vena cava leiomyosarcoma: what method of reconstruction for which type of resection?. World J Surg.

[3] Hardwigsen J, Balandraud P, Ananian P, Saïsse J, Treut YP (2005). Leiomyosarcoma of the retrohepatic portion of the inferior vena cava: clinical presentation and surgical management in five patients. J Am Coll Surg.

[4] Drukker L, Alberton J, Reissman P (2012). Leiomyosarcoma of the inferior vena cava: radical surgery without vascular reconstruction. Vasc Endovascular Surg.

[5] Puerta A, Vilar JA, Núñez J, Hervás PL, Nuño J (2020). Leiomyosarcoma of the inferior vena cava. Cir Esp.

[6] Mingoli A, Cavallaro A, Sapienza P, Marzo L, Feldhaus RJ, Cavallari N (1996). International registry of inferior vena cava leiomyosarcoma: analysis of a world series on 218 patients. Anticancer Res.

[7] Wang MX, Menias CO, Elsherif SB, Segaran N, Ganeshan D (2021). Current update on IVC leiomyosarcoma. Abdom Radiol.

[8] Mingoli A, Sapienza P, Cavallaro A (1997). The effect of extend of caval resection in the treatment of inferior vena cava leiomyosarcoma. Anticancer Res.

[9] Joung HS, Nooromid MJ, Eskandari MK, Wayne JD (2020). Surgical approach, management, and oncologic outcomes of primary leiomyosarcoma of the inferior vena cava: an institutional case series. J Surg Oncol.

[10] Nabati M, Azizi S (2018). Leiomyosarcoma of the inferior vena cava presenting as a cardiac mass. J Clin Ultrasound.

[11] Cho SW, Marsh JW, Geller DA (2008). Surgical management of leiomyosarcoma of the inferior vena cava. J Gastrointest Surg.

[12] Jiang H, Wang YX, Li B (2015). Surgical management of leiomyosarcoma of the inferior vena cava. Vascular.

[13] Daylami R, Amiri A, Goldsmith B, Troppmann C, Schneider PD, Khatri VP (2010). Inferior vena cava leiomyosarcoma: is reconstruction necessary after resection?. J Am Coll Surg.

[14] Hollenbeck ST, Grobmyer SR, Kent KC, Brennan MF (2003). Surgical treatment and outcomes of patients with primary inferior vena cava leiomyosarcoma. J Am Coll Surg.

