# Supplementary material for: Surgical resection of retrohepatic inferior vena cava leiomyosarcoma without vascular reconstruction: case report
Source: J Vasc Bras. 2023 Jul 10;22:e20220108. doi: 10.1590/1677-5449.202201081 (PMC10421572; doi:10.1590/1677-5449.202201081)
Supplement: Translation

# Translation: Ressecção cirúrgica de leiomiossarcoma de veia cava inferior retro-hepática sem reconstrução vascular: relato de caso

**DOI:** 10.1590/1677-5449.202201082

**Authors:** Ian Freire Castro, Paulo Henrique Silva Nunes, Ana Camila Xavier Lopes, Mariana Coelho Lima, Régis Ponte Conrado, Renato Mazon Lima Verde Leal, Annya Costa Araújo de Macedo Goes, Marcelo Leite Vieira Costa

**Affiliations:** 1 Hospital Geral de Fortaleza - HGF, Fortaleza, CE, Brasil.; 2 Universidade Federal do Ceará - UFC, Fortaleza, CE, Brasil.; 3 Hospital Universitário Walter Cantídio - HUWC, Fortaleza, CE, Brasil.

**Keywords:** leiomiossarcoma, veia cava inferior, neoplasias retroperitoneais, sarcoma, procedimentos cirúrgicos vasculares

## Abstract

O leiomiossarcoma de veia cava inferior (LVCI) é um raro tumor maligno mesenquimal. Seu tratamento cirúrgico é um desafio, pois necessita combinar margens cirúrgicas livres com reconstrução vascular, usando prótese ou enxerto autólogo, sutura primária ou ligadura simples sem reconstrução da veia. A ligadura é possível graças ao lento crescimento do tumor, permitindo o desenvolvimento de circulação venosa colateral. Apresentamos um caso de LVCI tratado por ressecção radical sem reconstrução vascular. Paciente feminina, 48 anos, com dor abdominal em hipocôndrio direito, astenia e sintomas dispépticos pós-prandiais. Tomografia de abdome revelou massa de formação expansiva localizada no segmento infra-hepático da veia cava inferior com redução da luz do vaso. Na cirurgia, o clampeamento da veia não indicou repercussões hemodinâmicas, sugerindo formação de circulação colateral suficiente. Decidiu-se pela ressecção radical em toda a porção da veia cava retro-hepática e ligadura da veia cava sem reconstrução vascular. A paciente evoluiu sem intercorrências.

## INTRODUÇÃO

O leiomiossarcoma de veia cava inferior (LVCI) é um raro tumor maligno mesenquimal oriundo da musculatura lisa venosa. Desde que foi descrito pela primeira vez em 1871 por Perl, foram documentados apenas um pouco mais de 400 casos em todo o mundo^1^ . Cerca de 2% de todos os leiomiossarcomas se originam em grandes vasos, com 60% deles na veia cava inferior (VCI)^2^ . Por apresentarem crescimento lento, permanecem assintomáticos por longos períodos, sendo diagnosticados acidentalmente em 10,5% dos casos^3^ .

Habitualmente, o LVCI produz manifestações clínicas tardias e inespecíficas. Os sintomas dependem de qual segmento da veia foi afetado. Dor abdominal leve e inespecífica é o sintoma mais prevalente, e raramente há massa abdominal palpável ao exame físico. As mulheres são mais frequentemente acometidas, e o pico de incidência ocorre na sexta década de vida. Ao diagnóstico, apresentam-se como grandes massas tumorais com tamanho médio de 10,8 cm^4^ . A ressecção cirúrgica do LVCI é um desafio, pois necessita combinar margens cirúrgicas livres com reconstrução vascular, usando prótese ou enxerto autólogo, sutura primária ou ligadura simples sem reconstrução da veia cava^2,5^ .

O presente artigo foi aprovado pelo Comitê de Ética em Pesquisa sob o parecer consubstanciado n° 5.560.148 (CAAE: 59402022.6.0000.5045).

## RELATO DE CASO

Uma paciente feminina, 48 anos, dona de casa, previamente hígida, buscou atendimento médico referindo sintomas dispépticos com piora pós-prandial, dor abdominal em hipocôndrio direito e astenia em 2016. Não havia história familiar de neoplasias. Ao exame físico, apresentava bom estado geral, com abdome doloroso à palpação e edema em membros inferiores. Ultrassonografia (USG) abdominal evidenciou massa retro-hepática com limites bem definidos. Após abandono de seguimento ambulatorial, foi encaminhada para nosso serviço em 2019, apresentando piora dos sintomas álgicos e dispépticos, com exceção do edema de membros inferiores, que havia cessado. A USG abdominal mostrou lesão com conteúdo anecoico bem delimitada no espaço hepatorrenal, medindo 10,5 x 7,3 cm. A tomografia computadorizada (TC) de abdome demonstrou massa de formação expansiva localizada no retroperitônio no segmento infra-hepático da VCI, com redução acentuada da luz (Figura 1). A biópsia hepática guiada por TC indicou neoplasia fusocelular de baixo grau histológico. A imuno-histoquímica revelou neoplasia de músculo liso de potencial maligno incerto (STUMP). A ressonância magnética mostrou dimensões estáveis em relação aos exames anteriores (Figura 2).

**Figura 1:** Imagem de tomografia computadorizada do abdome na fase arterial mostrando tumoração exercendo efeito de massa no fígado e acentuada redução da luz da veia cava retro-hepática.

**Figura 2:** (a) Ressonância magnética coronal em T2 mostrando leiomiossarcoma exercendo efeito de massa sobre a região do hilo hepático e obstruindo a veia cava inferior; (b) corte axial em T2 mostrando lesão com contornos regulares e limites bem definidos, com baixo sinal homogêneo, comprimindo a veia cava; (c) corte axial *dual echo* mostrando leiomiossarcoma em contato com os segmentos I, V e VI do fígado.

Na cirurgia, em maio de 2021, foi realizada incisão bicostal transversa associada a ampla manobra de Kocher e extensa mobilização do lobo direito do fígado, expondo todo o seguimento da cava abdominal, desde o diafragma até as veias renais (Figura 3). Na porção retro-hepática, visualizou-se a lesão obstruindo a luz do vaso, sem invasão do parênquima hepático ou de órgãos adjacentes (Figura 4). Após o clampeamento da veia, não foram observadas repercussões hemodinâmicas, indicando formação de circulação colateral suficiente decorrente da obstrução crônica. Decidiu-se pela ressecção do tumor em monobloco em toda a porção da veia cava retro-hepática (Figura 5). Os cotos da VCI foram suturados com grampos vasculares, e não foi realizada a reconstrução vascular do segmento seccionado. O exame anatomopatológico pós-operatório revelou leiomiossarcoma bem diferenciado, medindo 9,5 x 7,0 x 6,5 cm e pesando 281 g (Figura 6), de grau histológico tipo 1. Não foi realizado estudo genético da peça cirúrgica por indisponibilidade desse exame em nossa instituição.

**Figura 3:** Visualização do leiomiossarcoma após ampla mobilização do fígado e antes do clampeamento da veia cava acima e abaixo da tumoração.

**Figura 4:** Imagem do intraoperatório mostrando a veia cava inferior (seta) e o leiomiossarcoma (à esquerda) antes da ressecção cirúrgica.

**Figura 5:** Exérese da porção inferior da tumoração e hemostasia com grampos vasculares (seta).

**Figura 6:** Leiomiossarcoma após a ressecção cirúrgica.

A paciente evoluiu sem intercorrências e recebeu alta após 5 dias de pós-operatório. Dezessete meses após a cirurgia, apresentava-se estável e sem recidivas locais ou a distância (Figura 7). Segue em acompanhamento semestral em nosso serviço.

**Figura 7:** (a) Corte coronal de ressonância magnética em T2, realizado 17 meses após a cirurgia, evidenciando segmento da veia cava inferior seccionado e ausência de recidiva local; (b) imagem axial *dual echo* mostrando região retro-hepática livre de tumoração após a cirurgia.

## DISCUSSÃO

Anatomicamente, os LVCI podem ser classificados em três grupos: segmento I (infrarrenal), segmento II (entre as veias renais e as veias hepáticas) e segmento III (supra-hepático)^2,6^ . Os tumores do segmento II, como o descrito no presente relato, são os mais comuns e apresentam melhor prognóstico^6^ . Geralmente, manifestam-se somente com dor no hipocôndrio direito, mas podem mimetizar doenças do trato biliar e, na existência de envolvimento dos vasos renais, causar insuficiência renal^2^ .

O diagnóstico preciso é feito pelo exame histopatológico, que pode ser realizado por biópsia guiada por USG ou TC^1,7^ . É importante avaliar os diagnósticos diferenciais do LVCI, que incluem tumores que invadem a VCI, como carcinoma de células renais e as neoplasias retroperitoneais primárias, como leiomiomas, tumores adipocíticos, tumores fibroblásticos, rabdomiossarcomas e tumores císticos^7^ .

É essencial a ressecção circunferencial completa da VCI com margens negativas para minimizar o risco de recorrência local^2,4^ . Geralmente, a tumoração é frouxamente aderida às estruturas adjacentes e é limitada ao seu local de origem, o que permite sua dissecção com relativa facilidade. Nesses casos, a ressecção radical não requer mais de 1 cm de margem livre de veia cava^8^ . Se houver acometimento de órgãos adjacentes, é necessária a ressecção dos segmentos envolvidos^2^ .

Para a reconstrução da VCI, as opções incluem reparo primário, enxerto autólogo, enxerto protético ou ligadura simples sem reconstrução^9^ . A ligadura da veia é possível graças ao lento crescimento do tumor durante meses ou anos, permitindo o desenvolvimento de circulação venosa colateral^10^ . Em grandes ressecções em bloco, pode haver rompimento da rede venosa colateral, impossibilitando a ligadura da veia^11^ . A ligadura reduz o tempo operatório e evita as complicações associadas ao reparo por enxerto protético, como hemorragias, infecções e uso de anticoagulantes. Os principais riscos da ligadura da VCI incluem insuficiência venosa crônica das extremidades inferiores, insuficiência renal e edema de membros inferiores^2^ .

É fundamental definir qual paciente se beneficia ou não da ligadura da VCI. Nesse sentido, Jiang et al.^12^ propuseram critérios para a indicação da ligadura da cava com base na experiência de seu centro: (1) o curso da doença é superior a 1 ano, de modo que a circulação colateral possa ser suficiente; (2) pelo menos 75% da VCI está obstruída; e (3) no intraoperatório, a injeção intravenosa de 20 mg de furosemida leva a mais de 100 mL de urina em 30 minutos após o bloqueio temporário da VCI. Outra forma de avaliar a necessidade de reconstrução da VCI é a medida da pressão venosa na VCI após o pinçamento. A reconstrução pode ser necessária se a pressão exceder 30 mmHg^11^ . A presença de edema de membros inferiores no exame físico é um indício de formação venosa colateral insuficiente e torna a reconstrução da VCI obrigatória^2^ .

Segundo Daylami et al.^13^ , bons resultados clínicos podem ser alcançados sem reconstrução da VCI em tumores que surgem abaixo das veias hepáticas. Em seu estudo com seis pacientes, quatro possuíam VCI ocluída pelo tumor. Não foi realizada reconstrução da VCI em nenhum paciente. Após a cirurgia, três pacientes evoluíram com edema de extremidades, que foi bem tolerado e não causou sequelas permanentes. Nenhum dos pacientes apresentou recidiva local durante o acompanhamento por 30 meses^13^ . Hollenbeck et al.^14^ publicaram um estudo retrospectivo com 25 pacientes, dos quais 52% foram tratados com ligadura da VCI após a ressecção da tumoração. A morbidade pós-operatória mais comum foi edema de membros inferiores, mas nenhum paciente apresentou edema grave persistente. A ligadura da VCI acima das veias renais foi bem tolerada em pacientes com boa função renal pré-operatória e com trombose ou obstrução venosa suprarrenal associada a formação de circulação colateral^14^ .

Estudos mostraram que a radioterapia perioperatória está relacionada a aumento de sobrevida global quando comparada a cirurgia isolada em casos de sarcomas retroperitoneais. A radioterapia adjuvante também mostrou aumento da sobrevida em estudos específicos sobre LVCI e pode ser usada em pacientes com margens cirúrgicas positivas para reduzir as chances de recidiva local. A quimioterapia neoadjuvante e a radioterapia podem ser úteis em tumores irressecáveis para aumentar as chances de ressecção cirúrgica. Nesse contexto, os quimioterápicos mais utilizados são dacarbazina, doxorrubicina, ciclofosfamida e ifosfamida/cisplatina. Contudo, ainda não existe tratamento padrão de quimioterápicos para o LVCI, e as evidências de seu uso ainda são conflitantes. Atualmente, está sendo investigado o uso de bloqueadores do fator de crescimento semelhante à insulina tipo 1, inibidores de alvo da rapamicina em mamíferos e inibidores de poli ADP-ribose polimerase^3,7,9^ .

Após a ressecção tumoral, a taxa de sobrevida em 5 e 10 anos é de 49,4% e 29,5%, respectivamente, e a taxa de sobrevida livre de doença em 5 anos é de 6%^9^ . A recorrência local é encontrada em até 53% dos casos, e os locais mais frequentes de metástases são fígado, pulmões e ossos^4^ . O prognóstico está fortemente ligado à extensão da ressecção radical, à presença de margens cirúrgicas negativas, ao tamanho do tumor e à ausência de metástases no momento da cirurgia^7,9^ . Em caso de recidiva, a cirurgia continua sendo uma importante opção terapêutica, podendo melhorar a sobrevida. Nos casos em que a ressecção completa for impossível, a paliação pode ser feita com citorredução associada a radioterapia^1^ .

## CONCLUSÕES

Os LVCI são neoplasias malignas raras com apresentação clínica inespecífica. Seu diagnóstico depende de exames de imagens e confirmação por biópsia. A ressecção cirúrgica radical é a única modalidade terapêutica com possibilidade de cura. Em casos selecionados, não é necessário reconstruir a veia cava com próteses ou enxertos, em razão da formação de circulação venosa colateral que permite a realização da ligadura simples da veia com segurança. O papel das terapias neoadjuvantes e adjuvantes ainda não está bem estabelecido, e são necessários mais estudos para determinar o tratamento mais apropriado.
